# A method of identification and localization of tea buds based on lightweight improved YOLOV5

**DOI:** 10.3389/fpls.2024.1488185

**Published:** 2024-11-28

**Authors:** Yuanhong Wang, Jinzhu Lu, Qi Wang, Zongmei Gao

**Affiliations:** ^1^ Modern Agricultural Equipment Research Institute, Xihua University, Chengdu, China; ^2^ School of Mechanical Engineering, Xihua University, Chengdu, China; ^3^ Department of Biological Systems Engineering, Washington State University, Prosser, WA, United States

**Keywords:** tea buds, target detection, YOLOV5M-SBSD, lightweight modeling, deep learning

## Abstract

The low degree of intelligence and standardization of tea bud picking, as well as laborious and time-consuming manual harvesting, bring significant challenges to the sustainable development of the high-quality tea industry. There is an urgent need to investigate the critical technologies of intelligent picking robots for tea. The complexity of the model requires high hardware computing resources, which limits the deployment of the tea bud detection model in tea-picking robots. Therefore, in this study, we propose the YOLOV5M-SBSD tea bud lightweight detection model to address the above issues. The Fuding white tea bud image dataset was established by collecting Fuding white tea images; then the lightweight network ShuffleNetV2 was used to replace the YOLOV5 backbone network; the up-sampling algorithm of YOLOV5 was optimized by using CARAFE modular structure, which increases the sensory field of the network while maintaining the lightweight; then BiFPN was used to achieve more efficient multi-scale feature fusion; and the introduction of the parameter-free attention SimAm to enhance the feature extraction ability of the model while not adding extra computation. The improved model was denoted as YOLOV5M-SBSD and compared and analyzed with other mainstream target detection models. Then, the YOLOV5M-SBSD recognition model is experimented on with the tea bud dataset, and the tea buds are recognized using YOLOV5M-SBSD. The experimental results show that the recognition accuracy of tea buds is 88.7%, the recall rate is 86.9%, and the average accuracy is 93.1%, which is 0.5% higher than the original YOLOV5M algorithm’s accuracy, the average accuracy is 0.2% higher, the Size is reduced by 82.89%, and the Params, and GFlops are reduced by 83.7% and 85.6%, respectively. The improved algorithm has higher detection accuracy while reducing the amount of computation and parameters. Also, it reduces the dependence on hardware, provides a reference for deploying the tea bud target detection model in the natural environment of the tea garden, and has specific theoretical and practical significance for the identification and localization of the intelligent picking robot of tea buds.

## 1 Introduction

Tea is the second most consumed beverage globally; its unique aroma and characteristic flavor make it famous worldwide. With the booming economy of the tea market, the economic benefits of tea are also increasing. According to the statistical data report of the China Tea Circulation Association, in 2021, the total area of tea gardens in the country was 32640.6 $km^{2}$ , with an increase of 5.51% compared with that of 2020, of which the harvestable area was 29163.8667 $km^{2}$ , with a weighting of about 89.35%; the output was about 3,029,400 tons, with an increase of 2% compared with that of 2020; the total amount of domestic sales was 2,319,900 tons, with an increase of about 4.56%; total domestic sales amounted to 43,798,694,461.99 dollars, an increase of about 8.0% year-on-year; the average price of domestic sales was 19.02 dollars/g, an increase of 3.3% year-on-year. Tea buds with high nutritional value can be made into high-quality tea with high economic value. The plucking of tea buds must be graded and plucked on the tea buds, which are generally classified into three main categories: single bud, one bud and one leaf, and one bud and two leaves. The image of the tea bud classification is shown in **Supplementary Figure 1**.

With the globalization of the tea buds industry, countries worldwide are gradually researching the intelligent plucking of tea buds, especially for the target detection of tea buds, which has become a research hotspot. The tea buds’ target recognition methods can be roughly divided into three categories: traditional image processing algorithms based on color space, recognition methods based on traditional machine learning, and recognition methods based on deep learning (Bojie et al., 2019) achieved the segmentation of tea bud targets in tea bud images by extracting the RGB channels of the tea bud images and then performing HIS and HSV spatial conversion of the RGB color space, respectively, and calculating the channel component thresholds of the converted spatial model (Zhang et al., 2019) utilized the improved B-G algorithm for tea tree canopy processing to segment the tea bud image in the canopy image and then combined it with Bayesian discrimination to realize the recognition of tea buds and harvesting status. Under natural conditions, traditional image processing methods based on color or shape are difficult to perform well in natural and complex infield environments due to problems such as lighting and background complexity.

With the rapid development of machine vision technology, it has received more and more attention as it has demonstrated excellent capabilities in processing image features (Liu et al., 2019). (Karunasena and Priyankara, 2020) proposed a stacked class classifier based on the histogram of gradient features (HOG) combined with a support vector machine (SVM) for tea bud detection with an average detection rate of 55% (Wang et al., 2018) used the HIS model to identify and separate tea buds using the improved K-means algorithm after gray scaling the tea images with the S-factor. A comprehensive analysis of the traditional machine learning-based recognition method found that it relies on image pre-processing and data conversion; pre-processing is crucial, and if the processing is not reasonable, it will seriously impact the model’s accuracy. Secondly, the external environment dramatically affects the method and performs poorly in the natural complex tea garden environment.

Regarding the recognition of tea bud targets, most scholars use deep learning-based methods to realize the recognition of tea bud targets (Kamilaris and Prenafeta-Boldú, 2018; Chen and Chen, 2020) used Faster RCNN to identify the one tip with two leaves regions in tea bud images and then used the fully convolutional model FCN to identify the tea bud picking points in the one tip with two leaves regions (Yang et al., 2019) used an improved Yolo-V3 deep convolutional neural network to recognize tea bud picking points, combined with the K-means method to cluster the target box sizes and trained the model to recognize correctly up to more than 90% (Qian et al., 2020) proposed a tea bud segmentation method based on an improved deep convolutional coding network (TS-Segnet), and the segmentation results were approximately the same as the actual results (Yan et al., 2022) realized the recognition and localization of tea bud targets by building an improved Mask RCNN model (MR3P-TS model), and their experimental results showed that the picking point localization precision was 0.949 and the recall rate was 0.910. (Li et al., 2023) proposed a deep learning-based method for tea bud yield estimation by augmenting the YOLOV5 model with a squeeze and excitation network (SENet) and then combining the Hungarian matching algorithm and Kalman filtering algorithm to achieve reliable tea bud counts. The final results found that the model has an average accuracy of 91.88% (Chen et al., 2021) proposed a fresh tea bud detection method based on image enhancement fusion SSD (FTSD-IEFSSD). The authors used both the enhanced image and the original image for the detection sub-network through the image enhancement algorithm of RGB channel transformation, combined with multi-layer semantic fusion and adaptive score fusion, to nearly improve the target recognition accuracy (Gong and Wang, 2021) proposed an improved YOLOV4 tea bud target recognition method based on improved YOLOV4, and the final experimental results showed that the average accuracy of the model was 93.08% and the recall rate was 86.94%.

Currently, the main representative models for real-time target detection algorithms include RNN series [Faster RCNN (Ren et al., 2017), Mask RCNN (He et al., 2017)], YOLO series (V3 (Redmon and Farhadi, 2018), V4 (Bochkovskiy et al., 2020), V5), and DETR series [RTMDet (Lyu et al., 2022), DETRV2 (Chen et al., 2022)]. Considering the special growth environment and physiological characteristics of tea buds, we have chosen the single-stage object detection model YOLOV5 with faster detection speed as the benchmark model to meet the needs of real-time detection of tea buds. Meanwhile, most of the tea bud detection targets belong to regular growth. More research is needed on the multi-target detection of tea buds in complex environments. In contrast, most research has focused on improving the accuracy of tea bud detection for the tea buds detection model without considering the difficulty and cost of model deployment. Detecting tea buds in complex environments using lightweight models is a great challenge; this paper proposes a lightweight tea bud detection model (YOLOV5M-SBSD) for identifying tea buds in complex tea plantation environments, and the method proposed in this paper achieves fast real-time detection of tea buds.

## 2 Materials and methods

### 2.1 Data acquisition

In this study, the images of Fuding No. 4 white tea were collected on May 08, 2022, in the open tea garden of Chengdu Liangfeng Tea Co. in Pujiang County, Chengdu City, Sichuan Province, China (N: 30°09′56.45″ E: 103°23′49.90″)—Pujiang Liangfeng Tea Plantation as shown in **Supplementary Figure 2**. After a series of processing of the collected raw images, the final white tea dataset contained 5,287 images of tea buds, with a size of 960*1080 pixels, and saved in JPEG format. The iPhone 12 rear camera took the dataset’s original images, and the camera’s specific parameters are shown in **Table 1**.

**Table 1:** Camera Setup Parameters for collecting tea buds images.

Variable value	Status
Image size	1920*1080 Pixels
Flash	Off
Zoom	Off
Aperture	f/1.6
Exposure time	1/180 s
Focal distance	26mm
Operation	Manual
Macro	Off
Type	JPG

**Figure 1:**
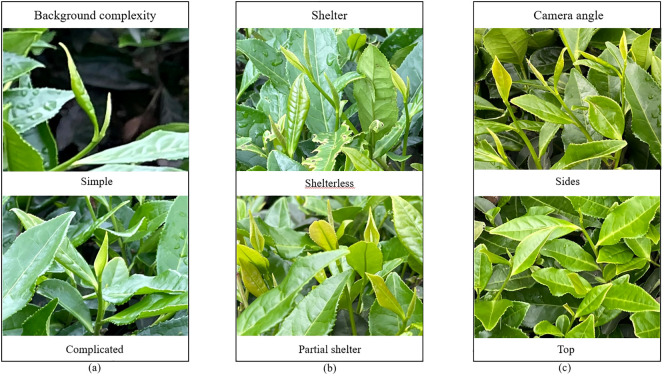
Acquisition of tea bud images under different conditions. **(A)** Tea bud background complexity; **(B)** Tea bud shelter; **(C)** Tea bud camera angle.

In this paper, when acquiring images, an acquisition device was used to capture images of tea buds at a distance of 300 mm-800 mm from the tea garden, and the shooting conditions included the background complexity **Figure 1A**, the shelter **Figure 1B**, and the camera angle **Figure 1C**, and an example of a sample image is shown in **Figure 1**.

In this paper, manually labeling is used to annotate the tea bud images to ensure the effectiveness of the annotation. Considering the quality of tea bud picking and the speed of tea bud positioning, we mainly focus on picking single buds and one bud with one leaf, and label their identification tags as burgeen uniformly. The tea buds with more than 2/3 occlusion are not annotated, and the XML file containing the coordinate information of the tea buds is generated after the annotation. The labeled dataset is divided into training set, validation set, and test set in the ratio of 8:1:1, and there is no repetition between each group.

### 2.2 Algorithm description of YOLOV5

**Figure 2:**
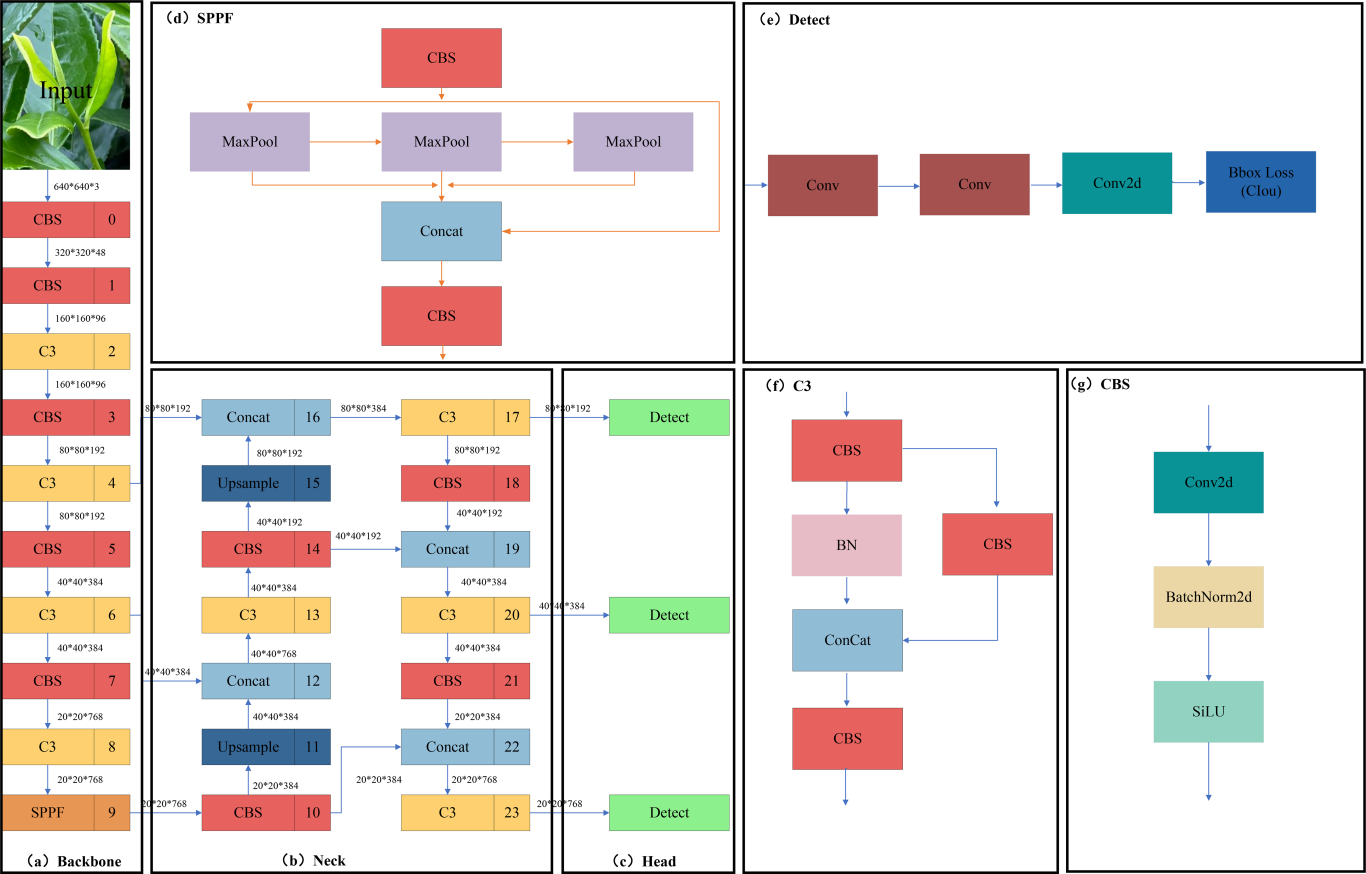
YOLOV5 network structure. **(A)** Backbone module; **(B)** Neck module; **(C)** Head module; **(D)** SPPF module; **(E)** Detect module; **(F)** C3 module; **(G)** CBS module.

The YOLOV5 model with relatively balanced accuracy and speed is selected in the first stage of the target detection algorithm model. However, the network width and depth will affect the training and detection time of the model, and there are four versions of the YOLOV5 model with differences in the network width and depth, namely YOLOV5S, YOLOV5M, YOLOV5L, and YOLOV5X. To meet the model’s lightweight deployment and real-time requirements and to consider image inputs of arbitrary size, YOLOV5M version 6.0 was finally selected as the benchmark model for Fuding white tea, and its network structure is shown in **Figure 2**.

The network structure of YOLOV5 is divided into the input side, as shown in the Backbone network **Figure 2A**, Neck network **Figure 2B**, and Head network **Figure 2C**. It mainly includes Mosaic data enhancement, adaptive anchor frame computation, and adaptive picture scaling at the input side; the Backbone mainly consists of the CBS module **Figure 2G**, C3 module **Figure 2F**, and SPPF module **Figure 2D**; Neck mainly consists of the CBS, up-sampling module (Upsample), Concat module, and C3; the Head mainly comprises three detection head Detect module **Figure 2E**.

The CBS mainly consists of a convolutional layer, batch-normalized (BN) layer, and sigmoid weighted linear unit (SiLU) activation function, in which the BN layer solves the problems of gradient vanishing and gradient explosion through data normalization. The SiLU activation function is a smooth and non-monotonic function that prevents the gradient from diminishing to 0 during the slow training process. C3 (Park et al., 2018) is a convolution module in YOLOV5, which serves to increase the receptive field of the network and improve the feature extraction capability of the network. The SPPF refers to a feature extraction module for target detection. The SPPF structure improves the model’s detection ability for targets of different sizes by pooling and fusing the feature maps of different sizes of receptive fields to obtain feature information of different scales. At the same time, the SPPF structure also has a specific downsampling effect, which can reduce the resolution of the feature map and improve the computational speed.

The Neck network is an intermediate feature extraction network added based on the Backbone, which is mainly used to enhance the feature expression ability and sensory field of the model to improve the detection performance of the model further. It mainly fuses the image features through the structure of the Feature Pyramid Network (FPN) and Path Aggregation Network (PAN) and transmits them to the detection end. The top-level semantic features are passed down through the top-down FPN (Lin et al., 2017), concatenating the lower and higher-level features using the bottom-up PAN (Liu et al., 2018). Finally, the feature information of different scales is fused, and the CIOU loss function is used at the output to measure the degree of gap between the predicted and natural frames. At the same time, the weighted non-maximum suppression (NMS) method is used for post-processing to remove the redundant candidate frames. The CIOU loss function (Zheng et al., 2022) increases the loss of the detection frame scale based on DIOU and increases the loss of the length and width, and the predicted frames are more in line with the actual frames, which improves the regression accuracy. The formula is shown in (Equation 1):


(1) $$\begin{array}{c} CIOU=IOU-\frac{\rho^{2}\left(b,b^{gt}\right)}{c^{2}}-\alpha v \end{array}$$

where $\rho^{2}\left(b,b^{gt}\right)$ represents the Euclidean distance between the centroids of the prediction and real frames, respectively, and c represents the diagonal distance of the smallest closure region that can contain both the prediction and real frames. The equations for α and v are shown in (Equations 2, 3):


(2) $$\begin{array}{c} \alpha =\frac{v}{1-IOU+v} \end{array}$$

(3) $$\begin{array}{c} v=\frac{4}{\pi^{2}}{\left(\tan^{-1}\frac{w^{gt}}{h^{gt}}-\tan^{-1}\frac{w}{h}\right)}^{2} \end{array}$$

At the prediction layer, CIOU_Loss is utilized to transfer the loss and weighted NMS is used to obtain the optimal target frame.

The model computation and complexity of the YOLOV5 algorithm in this study are too high, and the arithmetic power of agricultural embedded devices needs to be higher. Therefore, it is imperative to reduce the amount of computation, improve the detection speed, and ensure the detection accuracy.

### 2.3 Improving the YOLOV5 network design

**Figure 3:**
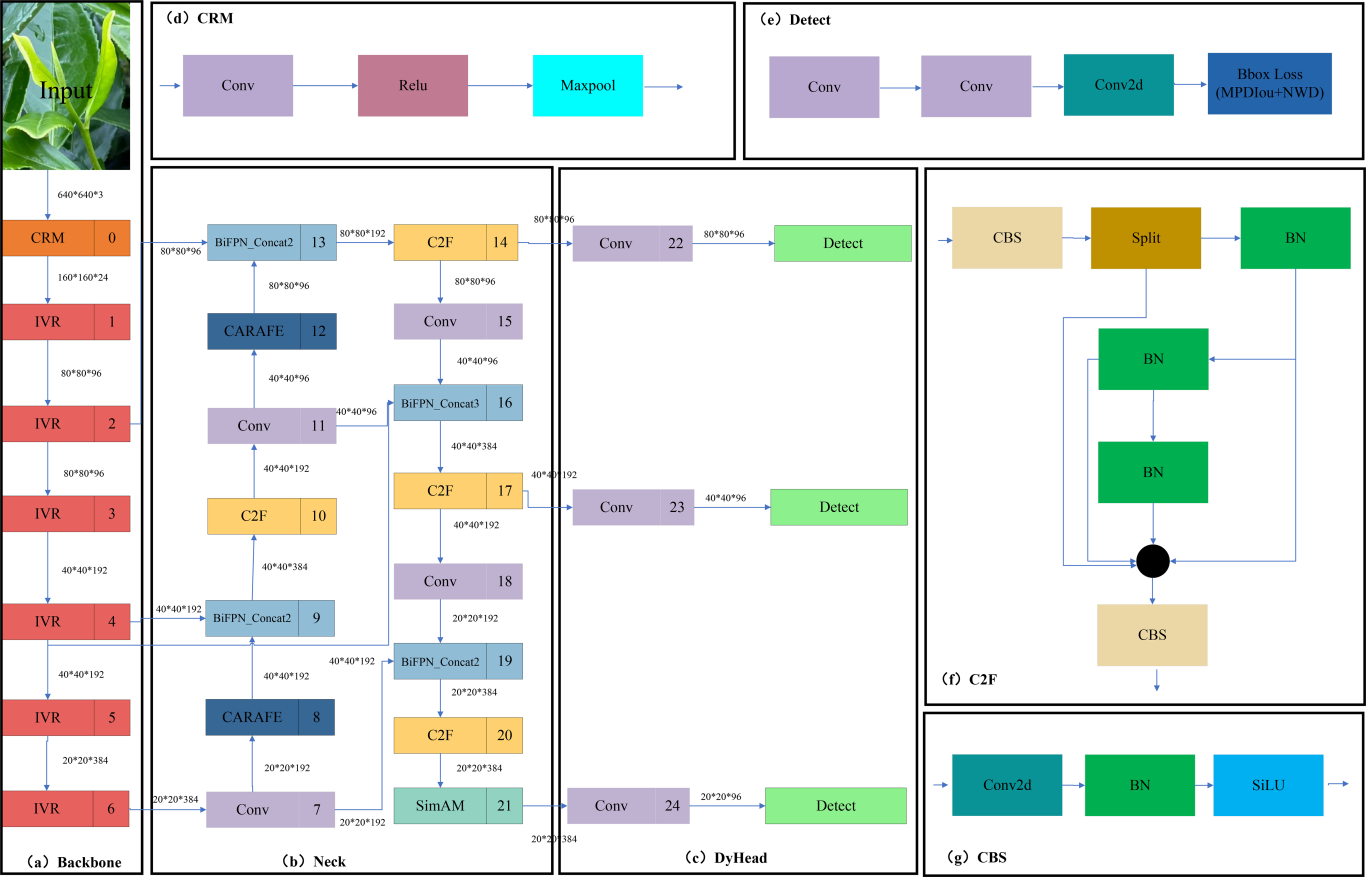
YOLOV5M-SBSD network structure diagram. **(A)** Backbone module; **(B)** Neck module; **(C)** DyHead module; **(D)** CBM module; **(E)** Detect module; **(F)** C2F module; **(G)** CBS module.

The YOLOV5M-SBSD network proposed in this paper consists of a Backbone network, Neck network, and Head network. The Backbone network replaces the original backbone network by utilizing ShuffleNetV2 (Ma et al., 2018), which reduces the computational effort and the number of parameters through channel rearrangement and group convolution. Channel rearrangement introduces cross-group connections in the network, reducing information transmission paths and improving feature interaction. Grouped convolution divides the input channels into multiple groups for convolutional operations, reducing the amount of computation in a single convolutional layer. The CARAFE (Wang et al., 2019) module structure was used in the Neck network to optimize the up-sampling algorithm of YOLOV5, which increases the sensory wildness of the network while maintaining lightweight. Due to the complex background environment of the tea plantation, significant differences in light intensity at different times and weather, inconsistent angles of the dataset, and other disturbing factors, the SimAM (Yang et al., 2021) attention module was embedded in the Neck network to improve the focusing on the tea bud target. To improve the accuracy of tea bud detection. In this study, SimAM is embedded into the tandem layer of PANet after giving higher weights to the semantic information of tea buds. Then, the C2F module replaces the C3 module in the Neck network. The C2F module can better adapt to targets of different sizes and shapes by using a variety of convolutional kernel sizes and step sizes, as well as a feature pyramid structure to capture feature information at different scales, improving the model’s detection capability and accuracy. The weighted bidirectional feature pyramid network (BiFPN) (Tan et al., 2020) replaces the PANet in the original model to achieve more efficient multi-scale feature fusion. In the Head network, we incorporate a dynamic target detection head (Dyhead) (Dai et al., 2021) to significantly improve the performance of the model target detection head without increasing the computational effort. Dyhead significantly improves the representation of the target detection head without any computational overhead by coherently combining the multi-head self-attention mechanism within the scale-aware feature layer, the spatial location for spatial awareness, and the output channel for task awareness. Finally, we replace the CIOU loss function of this model with MPDIOU (Ma and Xu, 2023) and incorporate the NWD (Wang et al., 2021) module, which can obtain faster convergence and more accurate regression results, effectively improving the detection accuracy and localization speed of the tea bud target. The YOLOV5M-SBSD network structure is shown in **Figure 3**.

#### 2.3.1 Backbone network improvements

**Figure 4:**
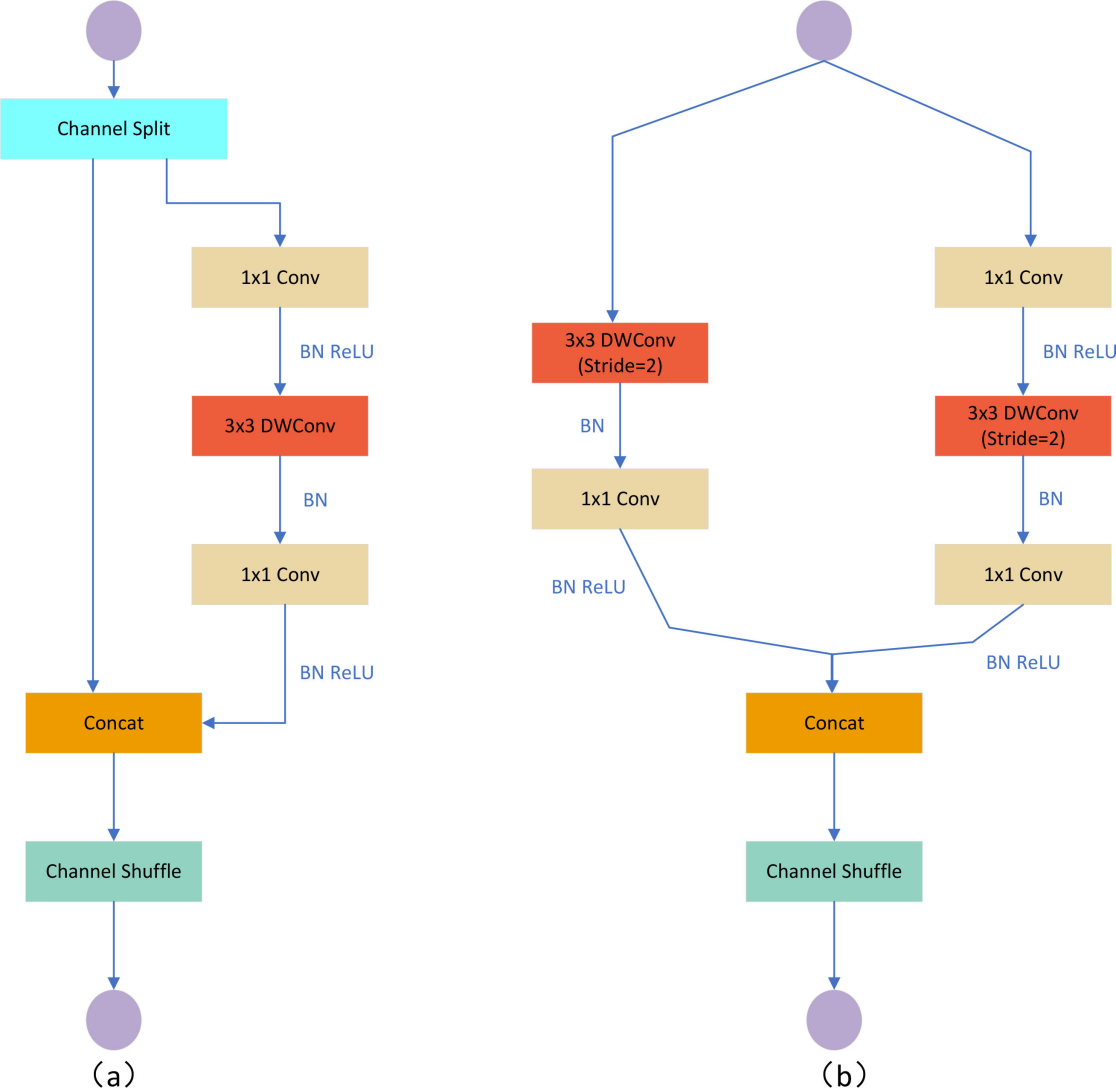
ShuffleNetV2 network structure diagram. **(A)** Basic unit; **(B)** Spatial downsampling unit DWConv depth convolution.

We replace the original backbone of YOLOV5M with the ShuffleNetV2 lightweight Backbone network, and the V2 version introduces a new operation, Channel Split. First, at the beginning, the input feature map is divided into two branches in the channel dimension, with the channel numbers C’ and C-C,’ and the actual implementation is C’= C/2. The left branch is mapped equally; the right branch contains three consecutive convolutions and has the same input and output channels, while the two 1x1 convolutions are not group convolutions; the two branches are equivalent to two groups. The output of these two branches will not be an Add element but a Concat operation for both branches. Then, Channel shuffle for the result of the Concat operation to ensure the exchange of information between the two branches. Moreover, the Concat and Channel shuffle can be combined with the Channel Split of the next module to form an element-level operation. Instead of having Channel Split, the downsampling module has one copy of the input for each branch, and each branch has a stride=2 downsampling. Finally, after Concat together, the feature map space size is halved, but the number of channels doubles. Meanwhile, V2 adds a Conv5 convolution before global Pooling, which differs from the V1 version. Under the same condition, ShuffleNetV2 is slightly faster and more accurate than other lightweight models. The network structure of ShuffleNetV2 is shown in **Figure 4**.

#### 2.3.2 Lightweight upsampling CARAFE module

**Figure 5:**
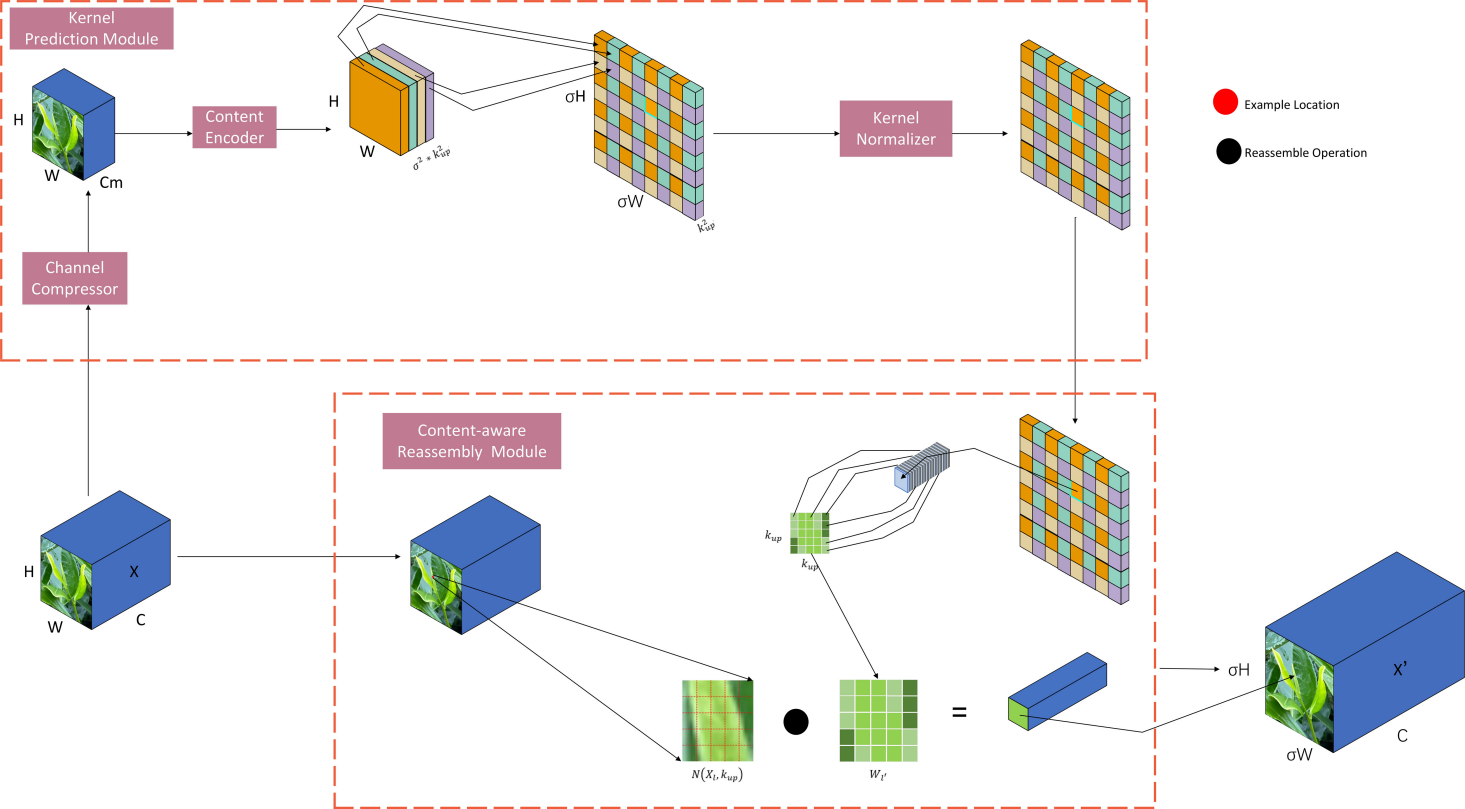
CARAFE network structure.

CARAFE is divided into two main modules, which are the up-sampling prediction module and feature reorganization module; assuming that the multiplicity of up-sampling is σ, given an input feature map with shape H*W*C, CARAFE firstly measures the up-sampling kernel by using the up-sampling prediction module and then completes the up-sampling by using the feature reorganization module, to get the output feature map with the shape of σH∗σW∗C , and the network structure diagram of CARAFE is shown in **Figure 5**.

In the up-sampling feature prediction module the feature map channels are first compressed, and for input shape feature maps with shapes such as H * W * C, the number of channels is compressed to Cm using 1 * 1 convolution to reduce the amount of computation in the subsequent steps. Then it is subjected to content encoding and up-sampling kernel prediction, assuming that the size of the up-sampling kernel is $k_{up}*k_{up}$ , and the amount of computation increases with the increase of the up-sampling kernel, and if we use a different up-sampling kernel for each position of the output feature map, then we need to predict the shape of the up-sampling kernel as $\sigma H*\sigma W*k_{up}*k_{up}$ , and the shape of the up-sampling kernel is $\sigma H*\sigma W*k_{up}*k_{up}$ , for the compressed input feature maps in the first step, utilizing a $k_{encoder}*k_{encoder}$ convolutional layer to predict the upsampling kernel, the number of input channels is Cm, the number of output channels is $\sigma^{2}K_{up}^{2}$ , and then the channel dimensions are expanded in the spatial dimension, and finally we get an upsampling kernel with the shape of $\sigma H*\sigma W*k_{up}^{2}$ , and then finally the upsampling kernel in the second step is subjected to a normalization operation using softmax to make the convolution kernel’s weights sum to 1. In the feature reorganization module, each position of the output feature map is mapped back to the input feature map, the $k_{up}*k_{up}$ region centered on it is taken out, and at the same time the up-sampling kernel of the point is predicted as a dot product, to get its output value, and different channels at the same position share the same up-sampling kernel.

#### 2.3.3 C2F module

We use the idea of a C2F module in YOLOV8 to replace the original C3 module; the C2F module is a stage Partial Network, which is used for feature fusion, and its central role is to fuse different levels of features to improve the performance of target detection.C2F module mainly consists of two parts, the upsampling and feature fusion two The up-sampling part matches the size of the high-level feature map by scaling the low-resolution feature map to high resolution through interpolation operation. The feature fusion part adds the up-sampled feature maps with the corresponding low-level feature maps element by element to fuse the semantic information of different levels. Through the operation of C2F, YOLOV5M-SBSD can fuse multi-level feature information while maintaining high resolution, thus improving the accuracy and robustness of the tea bud target detection, better capturing the detailed information of the target, and reducing the leakage and misdetection. The network structure of C2F is shown in **Supplementary Figure 3**.

#### 2.3.4 BiFPN module

**Figure 6:**
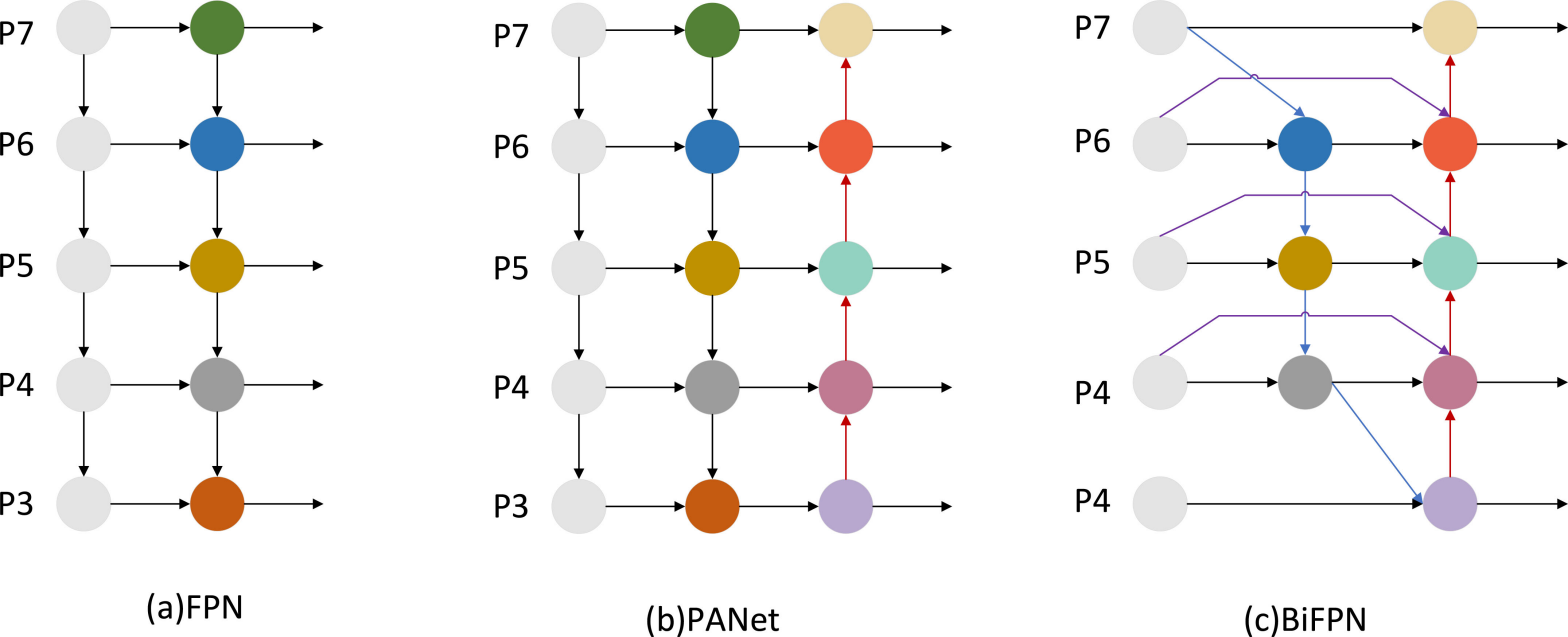
BiFPN network design. **(A)** FPN introduces a top-down path to fuse multi-scale features from P4 to P8; **(B)** PANet adds a bottom-up path on top of FPN; **(C)** BiFPN removes redundant nodes from PANet and adds additional connections.

We introduce a BiFPN in the Neck network to replace the original Concat layer. The BiFPN network structure is weighted and bidirectionally connected, i.e., top-down and bottom-up structures, and cross-scale connectivity is achieved by constructing bidirectional channels, which directly fuse the features in the feature extraction network with the relative-size features in the bottom-up paths, retaining shallower semantic information and less loss of deep semantic information. At the same time, BiFPN sets different weights according to the importance of different input features while repeatedly adopting this structure to enhance the feature fusion. The weighted fusion in the BiFPN structure adopts the fast normalized fusion, which is proposed for the slow training speed, and scales down the weights to the range of 0~1, and the training is fast because it does not use the Softmax method. The cross-scale connection is realized by adding a jump connection and a bi-directional path; the weighted fusion and bi-directional cross-scale connection have been realized. The structure of FPN is shown in **Figure 6A**, the structure of PANet is shown in **Figure 6B**, and the structure of BiFPN is shown in **Figure 6C**.

#### 2.3.5 SimAm attention mechanism

**Figure 7:**
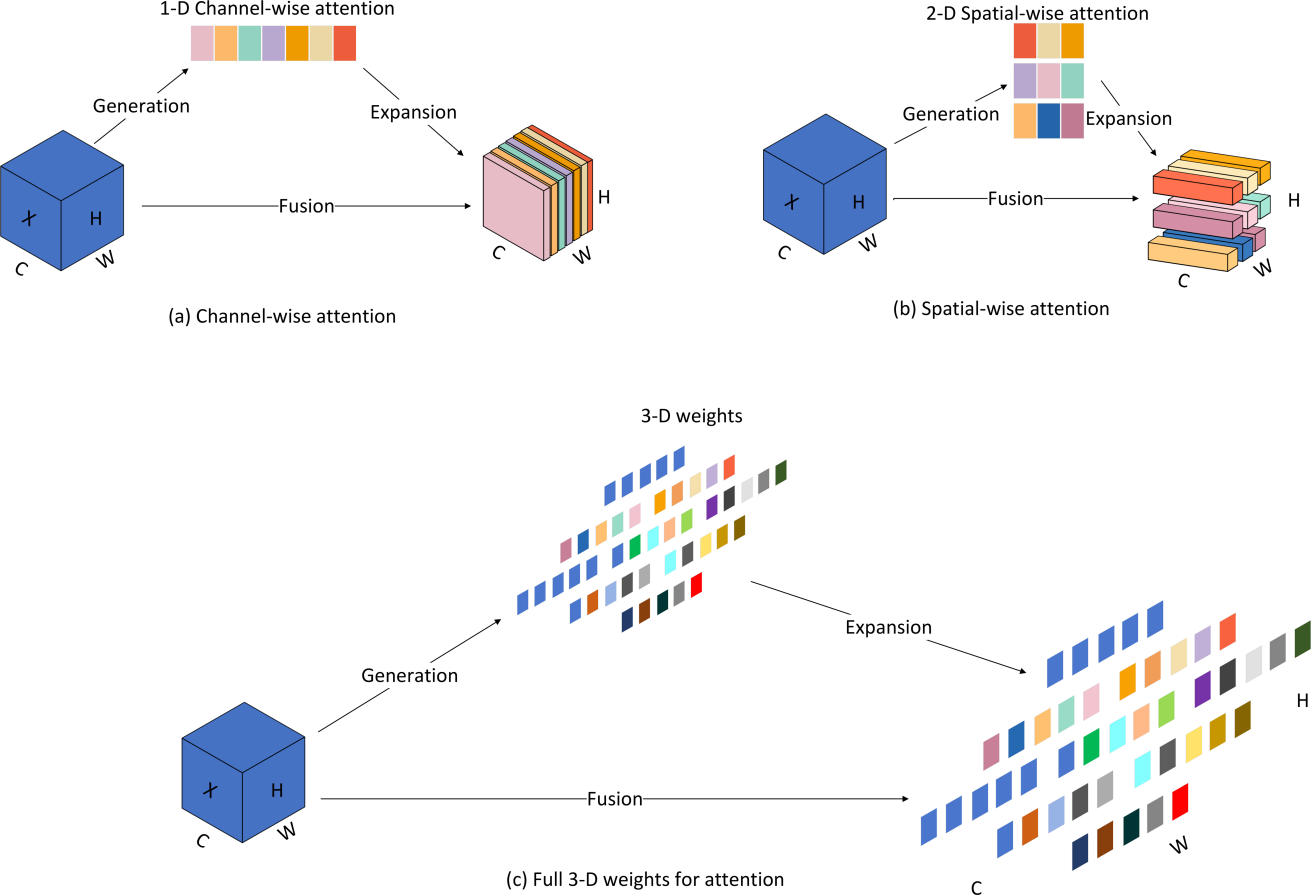
Comparison of weighted attention in different dimensions. **(A)** Channel-wise attention; **(B)** Spatial-wise attention; **(C)** Full 3-D weights for attention.

We introduce a parameterless attention mechanism, SimAm, in the new model, which is simple and efficient compared to other attention modules. Unlike existing channel or spatial attention modules, this module does not require additional parameters to derive 3D attention weights for the feature map. Currently, existing attention modules are usually inherited into each block to improve the output from previous layers. This refinement step usually operates along the channel dimension (**Figure 7A**) or the spatial dimension (**Figure 7B**), and these methods generate one- or two-dimensional weights and treat neurons at each channel or spatial location equally. Among them, channel attention belongs to 1D attention, which treats different channels differently and treats all locations equally. Spatial attention belongs to 2D attention, which treats different locations differently and treats all channels differently. SimAm belongs to 3D weighted attention (**Figure 7C**), which can learn more discriminative cues and is significantly better than traditional 1D and 2D weighted attention. Compared to other mainstream attention mechanisms, SimAm performs best and does not introduce additional parameters. The attention to different dimensional weights is shown in **Figure 7**.

#### 2.3.6 MPDIOU loss function

We introduced the MPDIOU loss function in YOLOV5M-SBSD to replace the original CIOU loss function. The MPDIOU loss function is a kind of bounding box regression loss function, which is used to measure the difference between the predicted box and the real box, and the use of MPDIOU can effectively solve the optimization problem of the bounding box regression loss function in the case that the predicted box and the actual labeled box have the same aspect ratio, but the width value and the height value are completely different. and height values are completely different, and can obtain faster convergence and more accurate regression results. MPDIOU is computed as follows, two arbitrary convex shapes: A, B ⊆ S ∈ Rn, the width and height of the input images are w, h. For A and B, $\left(x_{1}^{A},y_{1}^{A}\right),\;\left(x_{2}^{A},\;y_{2}^{A}\right)$ denote the coordinates of the upper-left and lower-right points of A, and $\left(x_{1}^{B},y_{1}^{B}\right),\;\left(x_{2}^{B},\;y_{2}^{B}\right)$ denote the coordinates of the upper-left and lower-right points of B. The MPDIOU is computed as follows.


(4) $$\begin{array}{c} d_{1}^{2}={\left(x_{1}^{B}-x_{1}^{A}\right)}^{2}+{\left(y_{1}^{B}-y_{1}^{A}\right)}^{2} \end{array}$$

(5) $$\begin{array}{c} d_{2}^{2}={\left(x_{2}^{B}-x_{2}^{A}\right)}^{2}+{\left(y_{2}^{B}-y_{2}^{A}\right)}^{2} \end{array}$$

(6) $$\begin{array}{c} MPDIOU=\frac{A\cap B}{A\cup B}-\frac{d_{1}^{2}}{w^{2}+h^{2}}-\frac{d_{2}^{2}}{w^{2}+h^{2}} \end{array}$$

This MPDIOU loss function is defined as follows:


(7) $$\begin{array}{c} L_{MPDIOU}=1-MPDIOU \end{array}$$

All factors of the existing bounding box regression loss function can be determined from the coordinates of the 4 points, and the conversion formula is shown below.


(8) $$\begin{array}{c} \left|C\right|=\left(\max\left(x_{2}^{gt},x_{2}^{prd}\right)-\min\left(x_{1}^{gt},x_{1}^{prd}\right)\right)\;*\;\left(\max\left(y_{2}^{gt},y_{2}^{prd}\right)-\min\left(y_{1}^{gt},y_{1}^{prd}\right)\right) \end{array}$$

(9) $$\begin{array}{c} x_{c}^{gt}=\frac{x_{1}^{gt}+x_{2}^{gt}}{2},\;y_{c}^{gt}=\frac{y_{1}^{gt}+y_{2}^{gt}}{2},\;y_{c}^{prd}=\frac{y_{1}^{prd}+y_{2}^{prd}}{2},\;x_{c}^{prd}=\frac{x_{1}^{prd}+x_{2}^{prd}}{2} \end{array}$$

(10) $$\begin{array}{c} w_{gt}=x_{2}^{gt}-x_{1}^{gt},\;h_{gt}=y_{2}^{gt}-y_{1}^{gt},h_{prd}=y_{2}^{prd}-y_{1}^{prd},w_{prd}=x_{2}^{prd}-x_{1}^{prd} \end{array}$$

In the above equation:

|C| denotes the minimum outer rectangle area covering Bgt and Bprd; $\left(x_{c}^{gt},y_{c}^{gt}\right)$ and $\left(x_{c}^{prd},y_{c}^{prd}\right)$ denote the coordinates of the centers of the real labeled bounding box and the predicted bounding box, respectively; $w_{gt}$ and $h_{gt}$ denote the width and height of the real labeled bounding box; $w_{prd}$ and $h_{prd}$ denote the width and height of the predicted bounding box.

**Figure 8:**
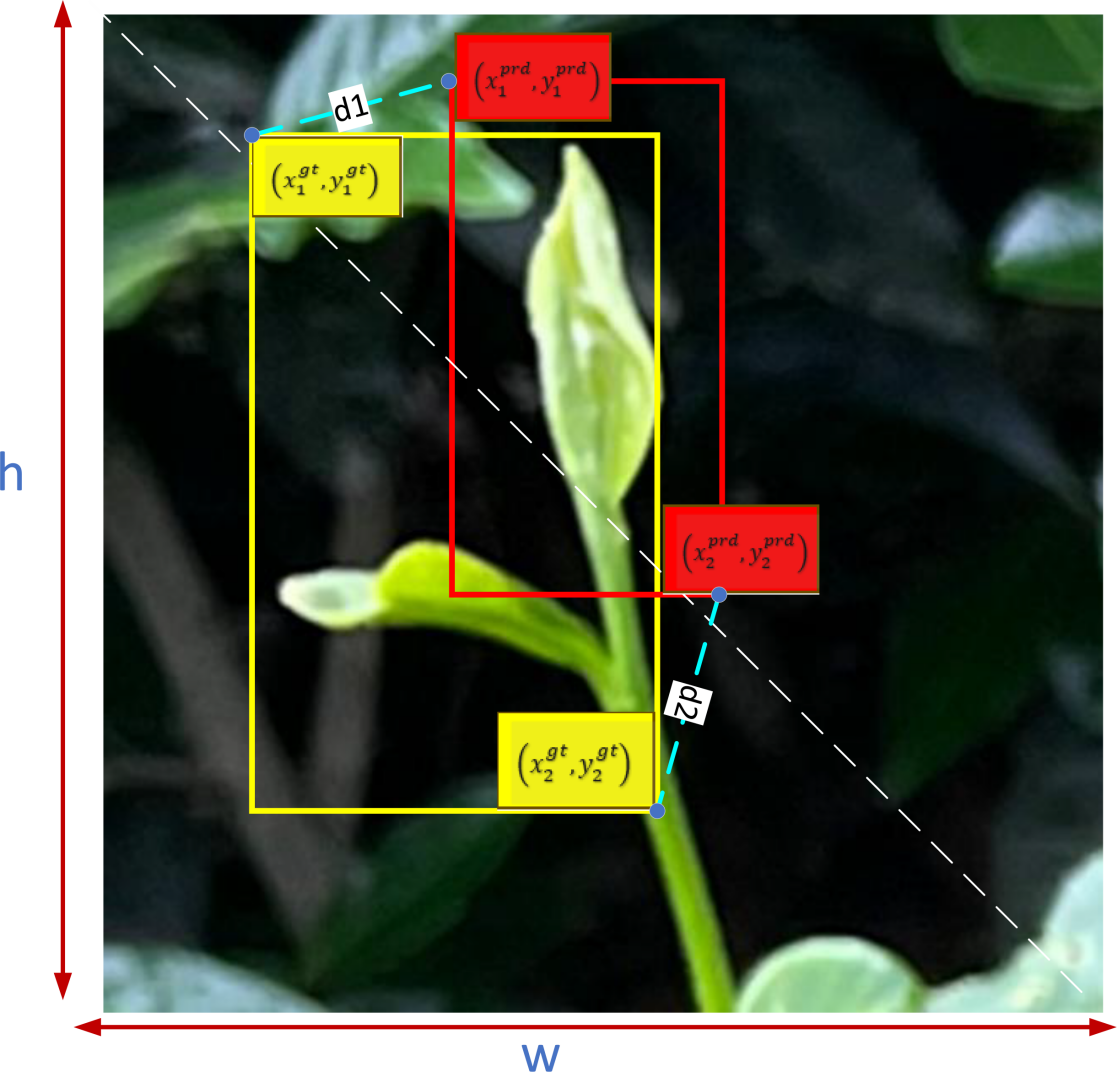
MPDIOU loss function.

The correlation diagram of the MPDIOU loss function is shown in **Figure 8**.

#### 2.3.7 Dyhead dynamic inspection head

We introduce the Dyhead dynamic detection head in our new algorithmic detection model, which significantly improves the representation of the target detection head without additional computational overhead by coherently combining the multi-head Self-attention mechanism within the scale-aware feature layer, spatial-aware spatial location, and task-aware output channel. By embedding Dyhead into the YOLOV5M-SBSD one-stage detection algorithm model, tea bud detection performance is significantly improved. The above three attention modules are defined as follows: for the scale-aware attention module, Scale-aware Attention (πL), which fuses features of different scales through semantic importance to enhance the scale-awareness of target detection.


(11) $$\begin{array}{c} \pi_{L}\left(F\right)*F=\sigma \left(f\left(\frac{1}{SC}\sum_{S,C}F\right)\right)*F \end{array}$$

where f(·) is a linear function, approximated with the use of 1*1 convolution; $\sigma \left(x\right)=\text{max}\left(0,\text{min}\left(1,\frac{x+1}{2}\right)\right)$ is a hard-sigmoid function.

For the spatial-aware attention module Spatial-aware Attention (πS), focusing on the discriminative ability of different spatial locations, deformable convolutional sparsification is first used. Then, the aggregated features of the feature layer are acquired at the exact location to enhance the spatial location awareness of target detection.


(12) $$\begin{array}{c} \pi_{S}\left(F\right)\;*\;F=\frac{1}{L}\sum_{l=1}^{L}\sum_{k=1}^{K}w_{l,k}*F\left(l;p_{k}+\Delta p_{k};c\right)*\Delta m_{k} \end{array}$$

where K is the number of sparsely sampled locations; $p_{k}+\Delta p_{k}$ does a location shift to focus on discriminative regions; and $\Delta m_{k}$ is a self-learnable importance metric factor with respect to location $p_{k}$ . All of the above features are obtainable by learning the input features from the F intermediate layer.

For the task-aware attention module Task-aware Attention (πC), different tasks are selected by dynamically turning on or off the feature channel to enhance the target detection’s ability to perceive different tasks.


(13) $$\begin{array}{c} \pi_{C}\left(F\right)*F=\max\left(\alpha^{1}\left(F\right)*F_{C}+\beta^{1}\left(F\right),\;\alpha^{2}\left(F\right)*F_{C}+\beta^{2}\left(F\right)\right) \end{array}$$

where ${\left[\alpha^{1},\alpha^{2},\beta^{1},\beta^{2}\right]}^{T}=\theta \left(\cdot \right)$ is the hyperfunction to control the activation thresholds; θ(·) first performs global pooling over the dimensions of L*S, then uses two fully-connected layers, a normalization layer, and finally normalizes the outputs using the Shifted sigmoid function.

**Figure 9:**
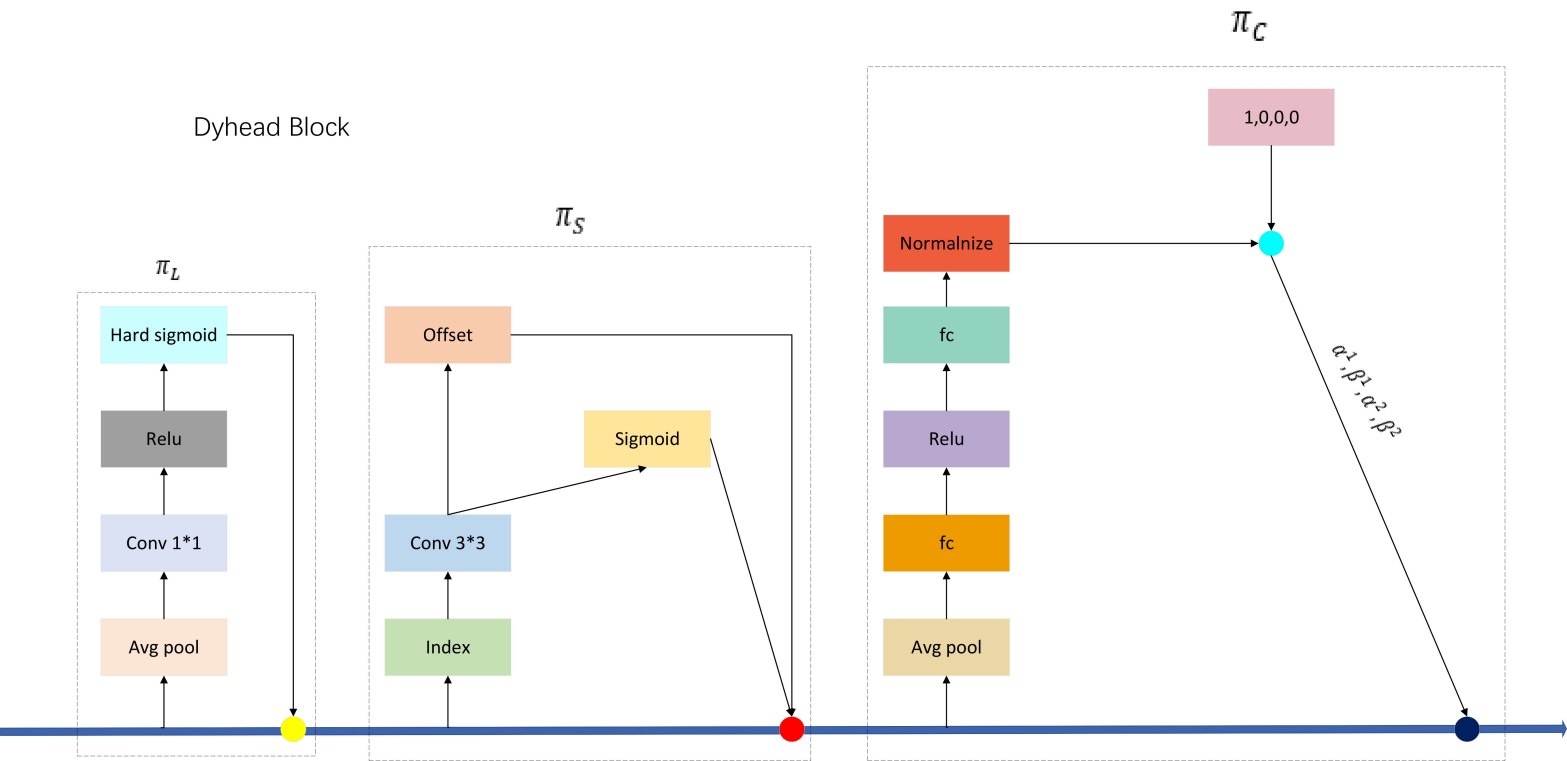
Dyhead network structure.

**Figure 10:**
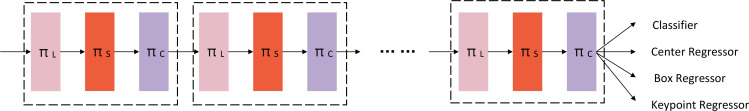
Dyhead embedded one-stage detection model.

The network structure of Dyhead is shown in **Figure 9**, and the structure of the embedded one-stage target detection model is shown in **Figure 10**.

### 2.4 Experimental environment and parameter configuration

This paper performs tests and runs on the same device, i.e., a desktop mainframe, with specific accessory configurations and experimental environments, as shown in **Table 2**.

**Table 2:** Hardware configuration and operating environment.

Hardware	Configure	Environments	Version
System	Windows10	python	3.10.11
CPU	Intel(R) i5-13400F	pytorch	1.13.1
GPU	NVIDIA RTX 3060 Ti	pycharm	2023.1
RAM	32.0 GB	CUDA	11.7
Hard-disk	1TB	CUDNN	8.4.1.5

Based on the hardware conditions, we set the hyperparameters with a learning rate of 0.01, momentum of 0.937, weight_decay of 0.0005, batch size of 16, workers of 2, and the optimizer uses stochastic gradient descent (SGD) and a single graphics processing unit (GPU) to accelerate training.

### 2.5 Evaluation indicators

The evaluation metrics used in this paper employ both performance and complexity; for the performance of the model, there are four metrics: Precision (P), Recall (R), Mean Accuracy (mAP), and F1-Score. P has been used to measure the performance of the model detection, and R has been used to assess the comprehensiveness of the detection (Hsu and Lin, 2021). The combination of P and R is the average precision (AP), and the average accuracy (map) is the mean value of AP, which is used to measure the performance of the whole model (Guo et al., 2022). mAP(0.5,0.95) denotes the mAP for different thresholds ranging from 0.5 to 0.95, in steps of 0.05. Since P and R are conflicting performance metrics, the F1-Score is the P and R, the reconciled mean of P and R with a range value of (0,1), which uses both P and R to assess the quality of the model. The specific formula is as follows: the quality of the model. The specific formula is as follows:


(14) $$\begin{array}{c} P=\frac{TP}{TP+FP}*100\% \end{array}$$

(15) $$\begin{array}{c} R=\frac{TP}{TP+FN}*100\% \end{array}$$

where TP is a positive sample predicted to be a positive class, FN is a positive sample predicted to be a negative class, and FP is a negative sample predicted to be a positive class:


(16) $$\begin{array}{c} AP=\int_{0}^{1}P\left(R\right)dR \end{array}$$

(17) $$\begin{array}{c} mAP\left(0.5\right)=\frac{\sum_{i=1}^{n}AP_{i}}{n} \end{array}$$

where n is the number of categories


(18) $$\begin{array}{c} F1=\frac{2*P*R}{P+R} \end{array}$$

For the model complexity aspect, there are three metrics: Params, GFlops and Size (Rampriya et al., 2022), which are formulated as follows:


(19) $$\begin{array}{c} Params=\left[r*\left(f*f\right)*o\right]+o \end{array}$$

(20) $$\begin{array}{c} GFlops=o\left(\sum_{i=1}^{n}k_{i}^{2}*C_{i-1}^{2}*C_{i}+\sum_{i=1}^{n}M^{2}*C_{i}\right) \end{array}$$

Where r is the input size, f is the convolution kernel size, o is the constant order, K is the convolution kernel size, C is the number of channels, M is the input image size and i is the number of iterations.

## 3 Results and discussion

### 3.1 YOLOV5M modeling improvement experiments

#### 3.1.1 Lightweighting module experiments

In this paper, YOLOV5M is chosen as the Backbone network, and its Backbone network is replaced by MobileNetV3, ShuffleNetV2, GhostNetV1, and GhostNetV2 for a comparison test. The careful consideration of the lightweight effect and the recognition accuracy finally determines the Backbone network of YOLOV5M-SBSD. It can be seen from **Table 3** that the comprehensive rate of the lightweight effect and the recognition accuracy of YOLOV5M-ShuffleNetV2 are significantly better than those of other types. Although the P and mAP of the benchmark model Backbone network are improved by replacing the Ghost series, the Params and GFlops are higher than those of MobileNetV3 and ShuffleNetV2 as the Backbone networks. After replacing the Backbone network of the original benchmark model with a lightweight network, the Params and GFlops of the tea bud detection model are reduced to a certain extent, while the P and mAP of the Ghost series are improved, the P and mAP of MobileNetv3 and ShuffleV2Net are reduced, and the R of the tea bud detection model is reduced to a certain extent. For the accuracy P of YOLOV5M-MobileNetV3, the mAP is 1.4% and 0.8% lower than those of the original benchmark model, and the number of Params, GFlops and Size are reduced by 89.99%, 91.65% and 89.16%, respectively; For the accuracy P of YOLOV5M- ShuffleNetV2, the mAP is 1.0% and 0.6% lower than those of the original benchmark model, and the number of Params, GFlops and Size were reduced by 90.31%, 91.03% and 89.51%, respectively; For the P and mAP of YOLOV5M-GhostNetV1 is 0.7% and 0.1% higher than those of the original benchmark model, and the number of Params, GFlops and Size were reduced by 59.19%, 62.11% and 54.73%, respectively; For the P and mAP of YOLOV5M-GhostNetV2 is 0.3% and 0.8% higher than those of the original benchmark model, and the number of Params, GFlops and Size were reduced by 4.05%, 10.44% and 3.49%, respectively. Considering the detection performance of the model, the detection accuracy and average accuracy of YOLOV5M-ShuffleNetV2 are better than those of YOLOV5M-MobileNetV3, and finally the Backbone network of YOLOV5M-SBSD is ShuffleNetV2.

**Table 3:** Comparison of YOLOV5M results under different backbone networks.

Model	P	R	mAP	Params/M	GFlops/G	Size/M
YOLOV5M	88.2%	89.3%	92.9%	20.852934	47.9	40.2
+MobileNetV3	86.8%	87.5%	92.1%	2.296612	4.0	4.76
+ShuffleNetV2	87.2%	87.9%	92.3%	2.020998	4.3	4.22
+GhostNetV1	88.9%	88.7%	93.0%	8.509854	18.2	16.8
+GhostNetV2	88.5%	88.3%	93.7%	20.009622	42.6	38.8

#### 3.1.2 Comparative experiments on attention mechanisms

After replacing the Backbone of YOLOV5M with ShuffleNetV2, the P and mAP are subsequently lost. Therefore, we consider introducing BiFPN to replace the original Concat layer of the model first and then introducing an attention mechanism to improve the recognition effect of YOLOV5M-ShuffleNetV2-BiFPN. This experiment adds CBAM, CA, ShuffleAttention, NAM, and SimAM attention mechanisms at the exact position of the Neck network of the YOLOV5M-ShuffleNetV2 base network to conduct comparative experiments. As seen from **Table 4**, the model’s detection is improved by embedding the attention mechanism, and the addition of all types of attention mechanisms except the SimAM attention mechanism leads to a slight increase in Params and GFlops. Although the improvement of CA for mAP is 0.1% higher than that of SimAM, its P is 1% lower than that of SimAM, and its Params, GFlops, and Size are 0.026616M,0.1G, and 0.05M higher than that of SimAM, respectively. Other types of attentional mechanisms have lower P and mAP than that of SimAM, and their Params, GFlops, and Size are higher than that of SimAM. They are all higher than SimAM. The introduction of the SimAM attention mechanism improves the P of YOLOV5M-ShuffleNetV2 by 0.4%. The experimental results show that using SimAM, a participantless attention mechanism, improves the feature extraction ability of the tea bud target, suppresses the interference of the complex background, and effectively improves the detection effect in detecting tea buds without increasing the complexity of the model.

**Table 4:** Comparison of YOLOV5M-ShuffleNetV2-BiFPN results with the addition of different attention mechanisms.

Model	P	R	mAP	Params/M	GFlops/G	Size/M
YOLOV5M+Sh+BiFPN	87.4%	87.1%	92.4%	2.057871	4.4	4.29
+CBAM	87.4%	87.4%	92.1%	2.076810	4.5	4.33
+CA	86.8%	88.3%	92.5%	2.084487	4.5	4.34
+ShuffleAttention	87.2%	87.7%	92.3%	2.061183	4.5	4.29
+NAM	87.6%	86.6%	92.1%	2.061807	4.5	4.29
+SimAM	87.8%	87.1%	92.4%	2.057871	4.4	4.29

#### 3.1.3 Comparative experiments on loss functions mechanisms

The introduction of parameter-free attention SimAM improves the detection effect of the target detection model on tea buds, and the loss function of the target detection model is replaced to improve the robustness of the training model. In this experiment, the EIOU, SIOU, WIOU, α_IOU, F_CIOU, F_EIOU, and MPDIOU loss functions were replaced for comparison experiments based on YOLOV5M-ShuffleNetV2-BiFPN-SimAM. From **Table 5**, it can be seen that different loss functions have no effect on the model complexity and have some effect on the P and mAP. The experimental results show that when the loss function is replaced with MPDIOU, the base P and mAP are increased by 0.1% and 0.2%, respectively, and nothing changes. The effect of other types of loss function relative to the base model loss function of the P have some reduction for the mAP, except for the loss function EIOU and WIOU remain unchanged, the rest of them are reduced than the base model. The MPDIOU loss function can obtain faster convergence speed and more accurate regression results.

**Table 5:** Comparison of YOLOV5M-ShuffleNetV2-BiFPN-SimAM results under different loss functions.

Model	P	R	mAP	Params/M	GFlops/G	Size/M
YOLOV5M+Sh+Bi+Si(CIOU)	87.8%	87.1%	92.4%	2.057871	4.4	4.29
+EIOU	87.6%	87.2%	92.4%	2.057871	4.4	4.29
+SIOU	86.8%	87.9%	92.2%	2.057871	4.4	4.29
+WIOU	87.4%	87.3%	92.4%	2.057871	4.4	4.29
+α_IOU	86.0%	86.8%	90.6%	2.057871	4.4	4.29
+F_CIOU	86.9%	88.2%	92.2%	2.057871	4.4	4.29
+F_EIOU	87.0%	86.9%	92.0%	2.057871	4.4	4.29
+MPDIOU	87.9%	87.1%	92.4%	2.057871	4.4	4.29

#### 3.1.4 Ablation experiments

We conducted ablation experiments on the lightweight module ShuffleNetV2, BiFPN, SimAM, loss function MPDIOU, C2F, NWD, lightweight Upsample CARAFE, and Dyhead, to evaluate the effectiveness of the YOLOV5M-SBSD detection algorithm in the detection of tea buds. It is worth noting that in order to make **Table 6** more aesthetically pleasing and concise, we have abbreviated the relevant modules in the ablation experiment results of **Table 6**, where Tag represents the serial number, Basic represents the baseline model, SNetV2 represents ShuffleNetV2, CAFE represents CARAFE, and Dhead represents Dyhead. From **Table 6**, it can be seen that a variety of model structures and algorithmic strategy-based improvement methods are effective, and compared with the original YOLOV5M, the P is improved by 0.5%, the mAP is improved by 0.2%, the Size is reduced by 82.89%, and the Params and GFlops are reduced by 85.6% and 83.7%, respectively.

**Table 6:** Ablation experiment results.

Tag	Basic	SNetV2	BiFPN	SimAM	MPIOU	C2F	NWD	CAFE	Dhead	P	mAP	Params/M	GFlops/G	Size/M
0	√									88.2%	92.9%	20.85293	47.9	40.2
1	√	√								87.2%	92.3%	2.020998	4.3	4.22
2	√	√	√							87.4%	92.4%	2.057871	4.4	4.29
3	√	√	√	√						87.8%	92.4%	2.057871	4.4	4.29
4	√	√	√	√	√					87.9%	92.4%	2.057871	4.4	4.29
5	√	√	√	√	√	√				87.9%	92.5%	2.057871	4.4	4.29
6	√	√	√	√	√	√	√			88.4%	92.9%	2.659599	5.7	5.43
7	√	√	√	√	√	√	√	√		88.4%	93.0%	2.793559	5.9	5.70
8	√	√	√	√	√	√	√	√	√	88.7%	93.1%	3.400287	6.9	6.88

**Figure 11:**
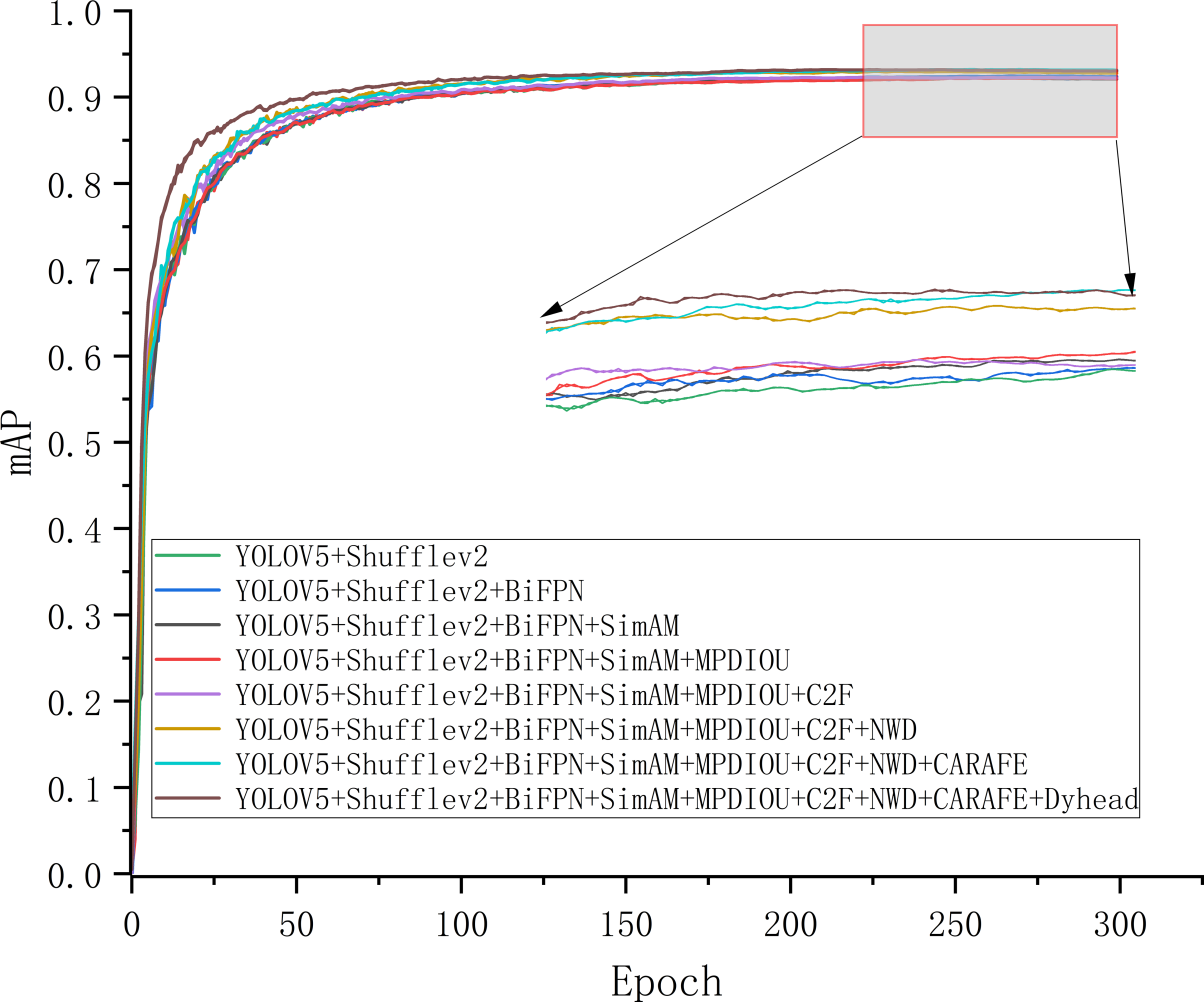
mAP curves for different stages of improvement.

Replacing the Backbone of YOLOV5M using Shufflev2Net significantly reduced the performance and complexity of the detection model, with the Size reduced by 82.89%, the Params and GFlops, reduced by 85.6% and 83.7%, respectively. However, the cost of changing the model to reduce the model complexity and computational volume was to reduce the effectiveness of tea bud identification, resulting in a 0.7% reduction in mAP. By replacing the original Concat layer with the introduction of BiFPN in the Neck network, the P was improved by 0.2%, and the mAP was improved by 0.1%, which improved the ability of mAP in the model to recognize tea bud targets. In addition, introducing the parameter-free attention mechanism SimAM without increasing the model complexity improves the P by 0.4%, and the mAP is unchanged. By replacing the loss function MPDIOU combined with NWD to achieve the optimization of the loss function, the P is improved by 0.6%, the mAP is improved by 0.5%, and finally, the Dyhead is replaced with the original detection head. Finally, the P is improved by 0.3%, and the mAP is improved by 0.2%. As shown in **Figure 11**, during the improvement process of the above method, the mAP is gradually improved, and the model complexity changes are minor, finally forming the YOLOV5M-SBSD detection model.

### 3.2 Experiments comparing the performance of different models

To verify the comprehensive performance of the YOLOV5M-SBSD model proposed in this paper, we used a total of Seven detection models: Faster RCNN, YOLOV3, YOLOV4, YOLOV4-tiny, YOLOV5S, YOLOV5M, and YOLOV5M-SBSD, for comparison, and the results of the performance tests are shown in **Table 7**.

**Table 7:** Comparison of experimental results for different models.

Model	P	mAP	Params/M	GFlops/G	Size/M
Faster RCNN	49.48%	84.02%	137.099	370.210	108
YOLOV3	88.2%	91.4%	61.497430	154.5	123.5
YOLOV4	73.7%	87.9%	52.496000	89.8	200
YOLOV4-tiny	67.6%	81.1%	5.874210	12.1	44.9
YOLOV5S	71.6%	70.9%	7.012822	15.8	14.4
YOLOV5M	88.2%	92.9%	20.852934	47.9	40.2
YOLOV5M-SBSD	88.7%	93.1%	3.400287	6.9	6.88

**Table 7** shows that the YOLOV5M-SBSD target detection model has the best performance in terms of precision P, mAP, Params, GFlops, and Size. The YOLOV5M-SBSD target detection model has the best performance in terms of P, mAP, Params, GFlops, and Size than Faster RCNN, YOLOV3, YOLOV4, YOLOV4-tiny, YOLOV5S and YOLOV5M. The P is 39.22%, 0.5%, 15%, 21.1%, 17.1% and 0.5% higher respectively, the mAP is 9.08%, 1.7%, 5.2%, 12%, 22.2% and 0.2% higher respectively, and the Params is 98.53%, 94.48%, 93.53%, 42.12%, 51.52% and 83.7% lower respectively, and the GFlops is 98.14%, 95.54%, 92.33%, 42.98%, 56.33% and 85.6% lower respectively, and the Size is 93.63%, 94.43%, 96.56%, 52.23%, 84.68%, and 82.89% lower respectively. The YOLOV5 version of the follow-up is widely used due to its flexibility, and the model complexity of YOLOV5M is high, which is not conducive to model deployment on low-computing-power devices, but after lightweight its detection performance will be reduced to a certain extent, so by making a series of improvements to the lightweight YOLOV5M to achieve its detection performance, which shows that YOLOV5M-SBSD has the best performance among the one-stage target detection algorithms.

### 3.3 Analysis of model detection effect

**Figure 12:**
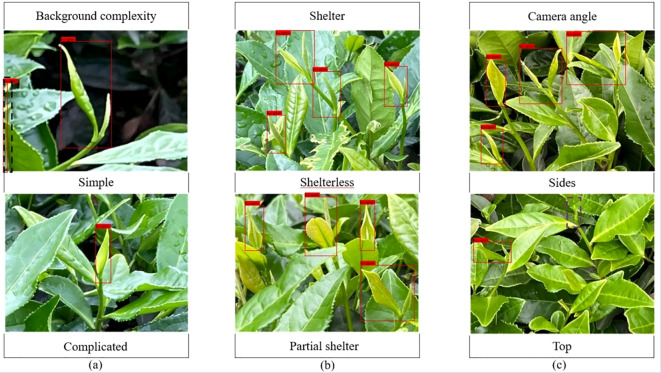
Faster RCNN detection accuracy. **(A)** Tea bud background complexity; **(B)** Tea bud shelter; **(C)** Tea bud camera angle.

**Figure 13:**
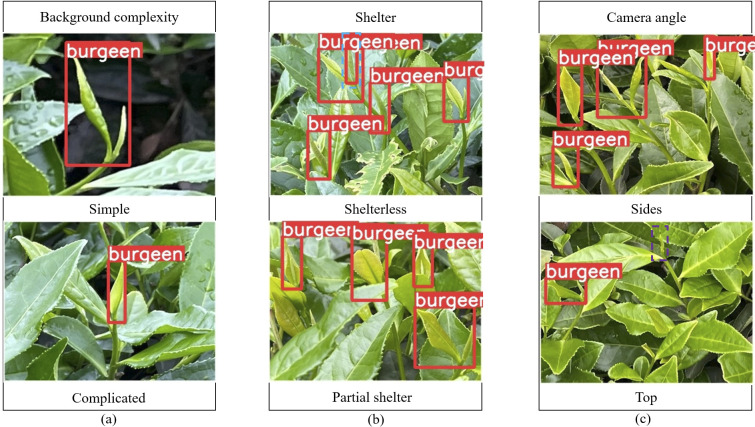
YOLOV3 detection accuracy. **(A)** Tea bud background complexity; **(B)** Tea bud shelter; **(C)** Tea bud camera angle.

**Figure 14:**
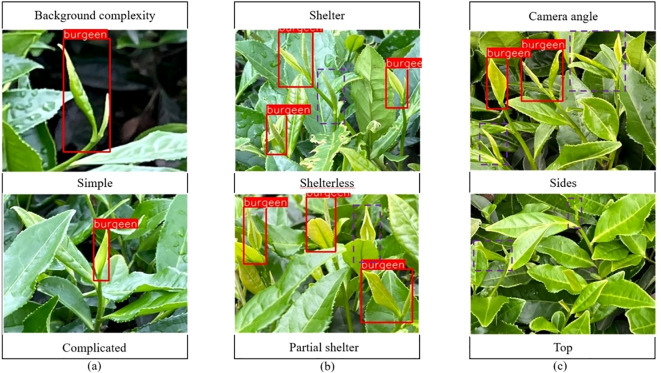
YOLOV4 detection accuracy. **(A)** Tea bud background complexity; **(B)** Tea bud shelter; **(C)** Tea bud camera angle.

**Figure 15:**
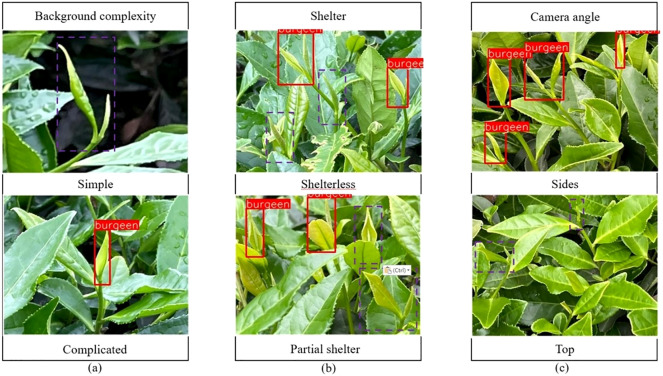
YOLOV4-tiny detection accuracy. **(A)** Tea bud background complexity; **(B)** Tea bud shelter; **(C)** Tea bud camera angle.

**Figure 16:**
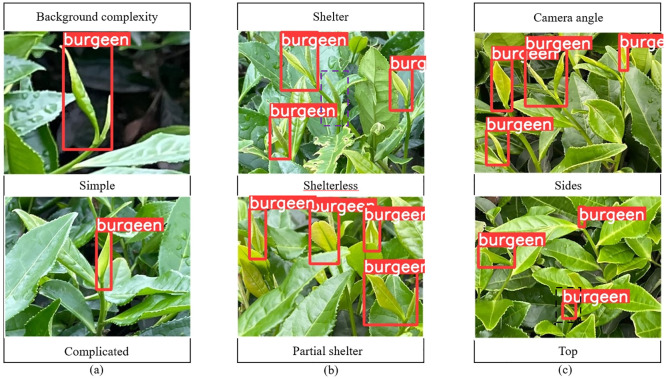
YOLOV5S detection accuracy. **(A)** Tea bud background complexity; **(B)** Tea bud shelter; **(C)** Tea bud camera angle.

**Figure 17:**
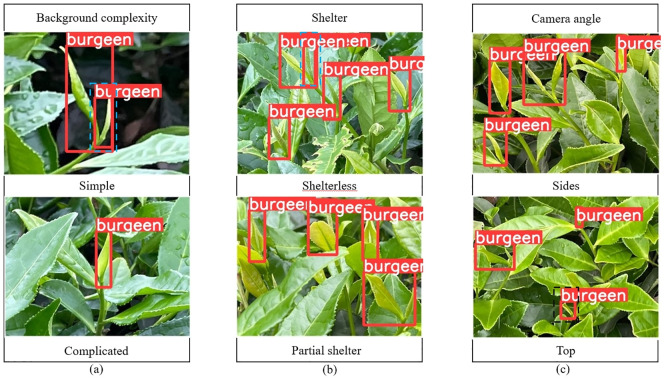
YOLOV5M detection accuracy. **(A)** Tea bud background complexity; **(B)** Tea bud shelter; **(C)** Tea bud camera angle.

**Figure 18:**
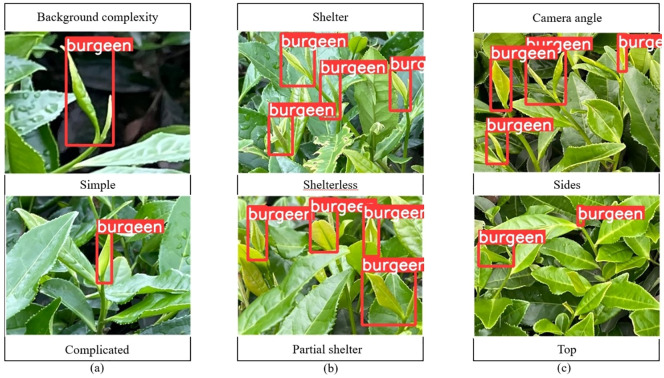
YOLOV5M-SBSD detection accuracy. **(A)** Tea bud background complexity; **(B)** Tea bud shelter; **(C)** Tea bud camera angle.

The improved model YOLOV5M-SBSD has a better overall performance for tea bud detection, and it is the best for different background complexity, different tea bud complexity, and different shooting angles; it has the lowest leakage and misrecognition rate, and the average detection rate of the model is above 80%, as shown in **Figures 12**–**18**. Among them, Faster RCNN, YOLOV3, YOLOV4, YOLOV4-tiny, YOLOV5S, and YOLOV5M have different degrees of misrecognition and even some leakage detection, and YOLOV4-tiny has the most severe leakage detection. In this research, black dashed boxes were utilized to indicate false detections, purple dashed boxes were used to indicate missed detections, and blue dashed boxes were used to indicate duplicate detections. For the Faster RCNN target detection algorithm, certain misdetections and omissions exist, such as Simple in **Figure 12A** and Top in **Figure 12C**. For the YOLOV3 target detection algorithm, certain duplicate identifications and omissions exist, such as Shelterless in **Figure 13B** and Top in **Figure 13C**. There are certain omissions for the YOLOV4 target detection algorithm, such as Shelterless and Partial shelter in **Figure 14B** and Top in **Figure 14C**. There are serious omissions for the YOLOV4-tiny target detection algorithm, such as Simple in **Figure 15A**, Shelterless and Partial Shelter in **Figure 15B** and Top in **Figure 15C**. The YOLOV5S target detection algorithm has certain misdetections and omissions, such as Top in **Figure 16C** and Shelterless in **Figure 16B**. The YOLOV5M target detection algorithm has certain duplicate identifications and misdetections, such as Simple in **Figure 17A**, Shelterless in **Figure 17B** and Top in **Figure 17C**. For our proposed YOLOV5M-SBSD target detection model, there is no such existing situation as mentioned above, which effectively illustrates the excellent performance of our proposed model.

### 3.4 Discussion

Visual recognition is a prerequisite for the intelligent picking of tea buds, which is of great significance for developing intelligent picking equipment for tea buds. With the advancement of computer technology and the development of agricultural robots, the application scope of deep learning has become more and more extensive. The single-stage target detection algorithm YOLO series has received extensive attention from scholars to meet the need for real-time detection and adapt to the real-time detection of intelligent picking equipment (Li et al., 2022) proposed a method to achieve real-time detection of tea buds using the YOLOV3SPP deep learning algorithm combined with channel pruning; they achieved this by adding a pyramid pooling module to the YOLOV3 model while combining the channel pruning algorithm and then fine-tuning the model, and through experiments, it was ultimately found that the size of the model and the detection time was reduced relative to the previous model by 96.81% and 59.62%. The detection speed of the compressed model is 15.9 fps, which is 3.18 times that of the original model (Zhang et al., 2023) proposed a ShuffleNetv2-YOLOV5Lite-E-based edge device detection method for one-bud and two-leaf tea. The final experimental results show that the file size of the improved model is reduced by 27% relative to the previous model, and the detection speed of the improved model is 3.2 times faster than the original YOLOV5 model (Cao et al., 2022) proposed a tea bud detection algorithm combining GhostNet and YOLOV5 by comparing the newly improved model with Faster RCNN, YOLOV5, and YOLOV5- lite correlation models, and the final experimental results showed that the target recognition accuracy of the newly improved model was improved by 1.31%, 4.83%, and 3.59%, respectively, concerning the compared models. The mAP of the YOLOV5M-SBSD target detection model proposed in this paper is 93.1%, and the average detection speed of a single image is 15.41ms, which meets the requirement of real-time detection. In addition, from **Table 7**, it can be seen that compared with Faster RCNN, YOLOV3, YOLOV4, YOLOV4 tiny, YOLOV5S, YOLOV5M and YOLOV5M-SBSD the model proposed in this paper has higher detection accuracy and average detection rate, as well as lower model volume, Params and GFlops. In order to distinguish the difference between the newly proposed lightweight detection model and other detection models, we took the initials of the main modules added by the improvement as the suffix, and renamed the newly proposed lightweight detection model. Therefore, we named the newly proposed lightweight tea bud detection model YOLOV5M-SBSD. Our newly proposed lightweight tea bud detection model, YOLOV5M-SBSD, has a wide range of application prospects, such as crop yield estimation and intelligent picking robot equipment development. The YOLOV5M-SBSD tea bud detection model can effectively adapt to the equipment with low computing power and reduce the impact of insufficient computing power on the detection effect of tea buds. In addition, the model can provide a new idea for the detection of other target crops, and provide technical support for target detection under low computing power equipment.

In future research, the tea bud detection model will be optimized through transfer learning by combining the characteristics of other representative tea varieties. In addition, multi-source information fusion methods are used to reduce the influence of factors such as solid light on tea buds and to improve the ability to extract features from tea buds. We try to collect images of tea buds of the same variety in different periods and make corresponding data sets to reduce the influence of the growth characteristics of tea buds on the identification of tea buds. Finally, the improved lightweight model is deployed to a low-computing-power device to carry out picking experiments in the complex environment of tea gardens to verify the excellent performance of the improved algorithm.

## 4 Conclusions

In order to achieve accurate detection of tea buds in the complex environment of limited computing power equipment and tea gardens, this paper proposes an improved target detection model YOLOV5M-SBSD. The experimental results show that YOLOV5M-SBSD outperforms the YOLOV5M target detection algorithm model, with Params, GFlops, and Size decreasing from 20.852934M to 3.400287M, from 47.9G to 6.9G, and from 40.2M to 6.88M, respectively. Params, GFlops, and Size are reduced by 83.7%, 85.6%, and 82.89%, respectively. Meanwhile, the P of the target detection model improves by 0.5%, and the mAP improves by 0.2%. Compared with other mainstream target detection models YOLOV3, YOLOV4, YOLOV4-tiny, YOLOV5S, YOLOV5M, and Faster RCNN, YOLOV5M-SBSD has the highest detection accuracy of 88.7%, the highest average detection rate of 93.1%, and the lowest Params, GFlops, and Size, respectively, of 3.400287M, 6.9G, and 6.88 M. This effectively demonstrates that YOLOV5M-SBSD can effectively and accurately detect tea buds in complex environments and on computationally underpowered devices, provide technical support for the development of intelligent picking equipment for high-quality tea, and promote the intelligent development of the high-quality tea industry.

## Data Availability

The original contributions presented in the study are included in the article/**Supplementary Material**. Further inquiries can be directed to the corresponding author.

## Supplementary material

Supplementary Figure 1 Classification image of tea buds. The blue box is single tea; the purple box is one leaf; the red box is two leaves.

Supplementary Figure 2 Pujiang Liangfeng Tea Plantation.

Supplementary Figure 3 C2F network structure. **(A)** C2F module; **(B)** CBS module.
