# Supplementary figures and images for: A method of identification and localization of tea buds based on lightweight improved YOLOV5
Source: Front Plant Sci. 2024 Nov 28;15:1488185. doi: 10.3389/fpls.2024.1488185 (PMC11634601; doi:10.3389/fpls.2024.1488185)

**Supplementary Material:**


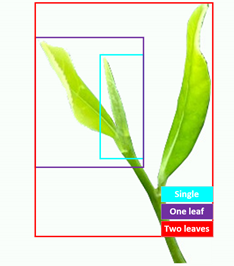


Supplementary Figure 1


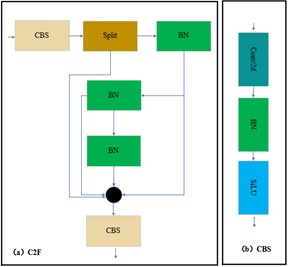


Supplementary Figure 2


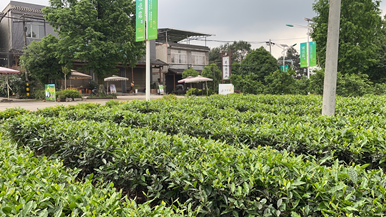


Supplementary Figure 3

Supplement: Supplementary Figure 1 — Classification image of tea buds. The blue box is single tea; the purple box is one leaf; the red box is two leaves. [file DataSheet1.docx]
